# Taiwanese Native Plants Inhibit Matrix Metalloproteinase-9 Activity after Ultraviolet B Irradiation

**DOI:** 10.3390/molecules14031062

**Published:** 2009-03-06

**Authors:** Yueh-Lun Lee, Mei-Hsien Lee, Hsiu-Ju Chang, Po-Yuan Huang, I-Jen Huang, Kur-Ta Cheng, Sy-Jye Leu

**Affiliations:** 1 Department of Microbiology and Immunology, Taipei Medical University, Taipei, Taiwan; 2 Graduate Institute of Pharmacognosy, Taipei Medical University, Taipei, Taiwan; 3 School of Nursing, Taipei Medical University, Taipei Taiwan; 4 Graduate Institute of Cell and Molecular Biology, Taipei Medical University, Taipei, Taiwan; 5 Applied Bioscience Division, Taiwan Sugar Research Institute, Tainan, Taiwan; 6 Department of Biochemistry, Taipei Medical University, Taipei, Taiwan; 7 Center for Reproductive Medicine and Sciences, Taipei Medical University Hospital, Taipei, Taiwan

**Keywords:** Taiwanese native plant, Polyphenolic, Matrix metalloproteinase-9.

## Abstract

Medicinal plants have long been used as a source of therapeutic agents. They are thought to be important anti-aging ingredients in prophylactic medicines. The aim of this study was to screen extracts from Taiwanese plant materials for phenolic contents and measure the corresponding matrix metalloproteinase-9 (MMP-9) activity. We extracted biological ingredients from eight plants native to Taiwan (*Alnus formosana*, *Diospyros discolor*, *Eriobotrya deflex, Machilus japonica, Pyrrosia polydactylis*, *Pyrus taiwanensis*, *Vitis adstricta, Vitis thunbergii*). Total phenolic content was measured using the Folin-Ciocalteu method. MMP-9 activities were measured by gelatin zymography. The extracted yields of plants ranged from 3.7 % to 16.9 %. The total phenolic contents ranged from 25.4 to 36.8 mg GAE/g dry material. All of these extracts (except *Vitis adstricta* Hance) were shown to inhibit MMP-9 activity of WS-1 cell after ultraviolet B irradiation. These findings suggest that total phenolic content may influence MMP-9 activity and that some of the plants with higher phenolic content exhibited various biological activities that could serve as potent inhibitors of the ageing process in the skin. This property might be useful in the production of cosmetics.

## Introduction

Medicinal plants have long been used as a source of therapeutic agents. Each plant’s extract contains different secondary metabolites. Polyphenols, which are common constituents, have also been reported to have antioxidant properties and the ability to inhibit the release of histamine [1] and various enzymatic activities [2,3,4,5,6], therefore, phenolics are thought to be health-promoting ingredients useful in prophylactic medicines. 

Matrix metalloproteinases (MMPs), a family of zinc-dependent endopeptidases, play a key role in the turnover of the extracellular matrix in skin. Aging and exposure to environmental insults, such as UV irradiation, increase the expression of MMP [6,7,8,9,10,11]. Excessive MMP activity, which causes the collapse of the meshwork in the extracellular matrix, produces UV irradiation-like skin damage, including wrinkling, loss of elasticity and dilation of surface micro-capillary vessels [12]. 

Some plants are considered natural active anti-aging agents for the skin. Because of Taiwan’s location and different altitudes, it has a very large array of plant species. The aim of this study was to screen plant material extracted from plants native to Taiwan for their phenolic contents and to investigate the *in vitr*o inhibitory effects of gelatinase (MMP-2 and -9) in human skin fibroblasts.

## Results and Discussion

The aim of this research was to examine the impact of phenolic contents on MMP-9 activity. The methodology employed herein is the Folin-Ciocalteu methid for phenolic compound extraction and gelatin zymography for determining MMP-9 activity. 

### Total phenolics contents in Taiwanese plants

Table 1 lists the eight Taiwanese native plants that were dissolved with 70% aqueous acetone, and the yields that were extracted from them (5.9 % to 27.3 %). Total phenolics were determined using the Folin-Ciocalteu method, and the contents were expressed as gallic acid equivalents (GAEs) per gram of dried plant extract. The eight plant extracts we measured ranged from 1.9 to 14.7 mg GAE/g dry material. The extracts from four species, *Diospyros discolor* Willd, *Eriobotrya deflex* (Hemsl.) Nakai, *Machilus japonica* Sieb. & Zucc. var. *kusanoi*, and *Pyrus taiwanensis* were rich in phenolics. 

The Folin-Ciocalteu method is a fast and simple screening method used routinely in our lab to measure total phenols. However, some interfering compounds, e.g. organic acids, sugars and amino acids are known to react with the reagents, which leads to the overestimation of the phenolic content. Therefore, compounds with bioactivity will be further isolated and characterized in a future study.

### Effect of Taiwanese Plants on cell viability on WS-1 cell

The cell viability for each native Taiwanese plant on human fibroblast WS-1 cell was assayed by the 3-[4,5-dimethylthiazol-2-yl]-2,5-diphenyl tetrazolium bromide (MTT) assay. The 200 μg/mL plant extracts were used for the test initially, however, if the cell viability was less than 50%, then 100 μg/mL or 10 μg/mL plant extracts were used. As shown in Table 2, at the added concentration none of the extracts affected WS-1 cell viability with or without UVB irradiation.

**Table 1:** Total phenolics content in Taiwanese Plants.

Botanical name; Family	Voucher specimen	Yield (%)	Total phenolics (mg of GAE /g)
*Alnus formosana* (Burk.) Makino; Betulaceae	M54	10.2	15.6
*Diospyros discolor* Willd.; Ebenaceae	M47	13.7	36.8
*Eriobotrya deflex* (Hemsl.) Nakai; Rosaceae	M50	8.8	29.6
*Machilus japonica* Sieb.＆Zucc. var. *kusanoi* (Hayata) Liao; Lauraceae	M67	12.8	25.4
*Pyrrosia polydactylis* (Hance) Ching; Polypodiaceae	M61	16.9	21.4
*Pyrus taiwanensis* Iketani ＆ Ohashi ; Rosaceae	M55	8.9	30.5
*Vitis adstricta* Hance; Vitaceae	M80	9.2	4.8
*Vitis thunbergii* Sieb. & Zucc; Vitaceae	M81	3.7	11.2

**Table 2:** Effect of Taiwanese Plants on cell viability on WS-1 cell.

Specimen	[μg/mL]	without UVB (%) mean ± SEM	[μg/mL]	with UVB (%) mean ± SEM
M54	[100]	90.45 ± 10.85	[100]	92.10 ± 2.20
M47	[100]	98.10 ± 1.10	[200]	109.7 ± 19.24
M50	[100]	103.9 ± 7.12	[100]	73.4 ± 23.4
M67	[100]	117.3 ± 14.7	[100]	140.3 ± 26.3
M61	[100]	94.4 ± 10.63	[200]	75.9 ± 13.5
M55	[10]	94.43 ± 5.42	[200]	81.25 ± 8.65
M80	[100]	128.1 ± 13.24	[100]	136.0 ± 6.20
M81	[100]	130.8 ± 14.96	[100]	109.1 ± 33.95
RA	[100]	112.7 ± 6.03	[100]	122.7 ± 1.95

RA: retinoic acid; Note: Averaged number of three experiments

### Effect of Taiwanese Plants on MMP-2 and 9 activities on WS-1 cell

**Figure 1:**
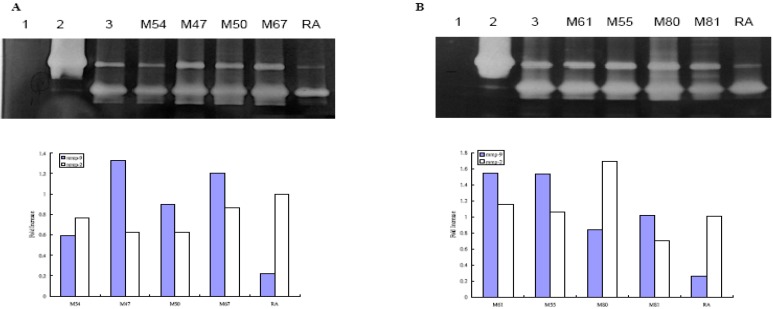
Effect of Taiwanese native plants on MMP-2 and –9 activities by zymography gel.

Figure 1 shows the MMP-9 activity measured by gelatin zymography. Of the eight Taiwanese plants we tested, two, namely *Alnus formosana* (Burk.) Makino and *Vitis thunbergii* Sieb. & Zucc, showed different degrees of inhibition of MMP-9 activity than the medium alone. Total phenolic amount did not correlate to the inhibition of MMP-9 activity. 

### Effect of Taiwanese Plants on MMP-2 and -9 activities after UVB irradiation on WS-1 cell

**Figure 2:**
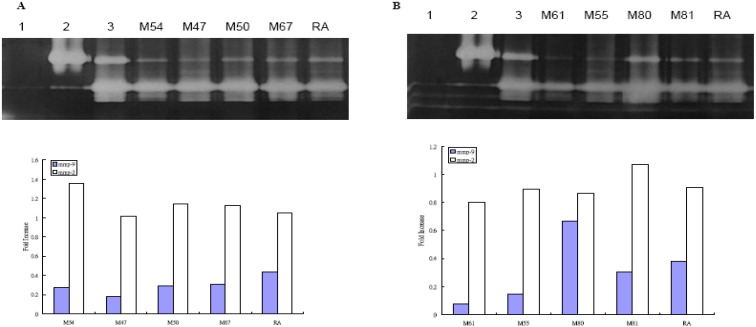
Effect of Taiwan native plants on MMP-2 and –9 activities by zymography gel after UVB irradiation.

Figure 2 shows the MMP-9 activity measured by gelatin zymography on WS-1 cells after UVB irradiation. All eight plants were found to suppress MMP-9 activity more than medium alone and the total phenolic amount correlated well with the inhibition, as the plant extract with the highest total phenolic content (*D. discolor*) showed the greatest inhibition of MMP-9 activity (Figure 2). Except for *Vitis adstricta* Hance, which had the lowest total phenolic count, the plants’ ability to inhibit MMP-activity was equal or better than the positive control, retinoic acid.

A and B represent MMP-2 and -9 activities of indicated Taiwanese native plants. Lanes 1-3 are negative controls, rMMP-9, medium alone without treatment, respectively. The lower panel represents the semiquantitation the MMP-2 and –9 activities by densitometer (gray and white bars represent MMP-9 and MMP-2 activities, respectively).

A and B represent MMP-2 and -9 activities of indicated Taiwanese native plants with UVB irradiation. Lanes 1-3 represents negative control, rMMP-9, medium alone without treatment, respectively. The lower panel represents the semiquantitation the MMP-2 and –9 activities by densitometer (gray and white bars represent MMP-9 and MMP-2 activities, respectively).

The potential of plant products in treating various diseases is often investigated. Phenolics, important secondary metabolites in plants, have been evaluated for radical scavenger activity [13,14,15] and their ability to inhibit lipid peroxidation [16,17,18,19]. Our study measured the phenolic content of eight Taiwanese plants and quantified their ability to inhibit MMP-9 activity in human skin fibroblast WS-1 cells. *D. discolor*, *E. deflex*, *M. japonica* var. *kusanoi*, and *P. taiwanensis* were found to have the greatest total phenolic content. We also found these four plants with the high phenolic content to clearly inhibit MMP-9 activity (Figure 1), but not MMP-2 activity after UVB irradiation (Figure 2). In the literature, the inhibition of MMP-9 activity has been compared with that of retinoic acid, which has the benefit of improving age-related skin damage [20,21,22]. The findings of our study showed that after UVB irradiation, all the native plants extracts, except *V. adstricta*, clearly inhibited MMP-9 activity better than retinoic acid. With or without UVB irradiation, *A. formosana* and *V. thunbergii* inhibited the MMP-9 activity (Table 2). However, since these two plants do not have high concentrations of phenolics, their ability to prevent skin damage may be due to the other secondary metabolites, such as terpenoids, alkaloids, or steroids. The eight plants used in this study are native to Taiwan. Except for *E. deflex,* which has been reported to have the free radical scavenging activities [23], the plants in this study have rarely been studied. Therefore, their major compounds need further identification and investigation.

Both UV irradiation and aging process, lead to increased ROS production, which in turn alters gene and protein structure and function. Thus, the aging process of the skin is associated with AP-1 activity, which results in increased MMP expression, impaired TGF-β signaling, enhanced collagen degradation, and decreased collagen synthesis [24]. Excess AP-1 activity can cause a collapse of the meshwork in the extracellular matrix to produce the visible effects of UV damage-- wrinkling, loss of elasticity and dilation of surface micro-capillary vessels. Several MMPs, (including MMP-1, -2, -3, -8, -9) have been found in greater quantities after UV irradiation [6, 9, 10, 25,26,27]. Therefore, MMP inhibitors may be used to protect the skin against environmental insults by developing effective cosmetic formulations that aim at: (1) delaying the appearance of fine lines and sagging skin, (2) reducing damage caused by exposure to the sun, (3) reducing skin redness, (4) reducing the appearance of spider veins (talangiectases), (5) reducing the appearance of dark circles around the eyes, (6) enhancing ECM cohesion, (7) improving the natural protective functions of the skin (against pollution, stress, age, sun, etc.), and (8) improving skin firmness and elasticity [28]. Thus, MMP might serve as a potential therapeutic target in treatment of aging. The inhibition of MMP may provide a strategic goal in cosmetology and may be used along with natural mechanisms to maintain healthy skin. Since the native Taiwanese plants extract we studied might serve as MMP inhibitors after UVB irradiation, they are well suited as potential sun-care products and can be used as protectors against environmental insults.

## Conclusions

In summary, the present investigation has shown the native Taiwanese plants inhibit MMP-9 activity after UVB irradiation. Therefore, they might contribute to the anti-aging effect of the human skin fibroblasts and they merit consideration a potential new ingredients in natural cosmetics. 

## Experimental

### Plant materials

All plant materials were obtained from the Taiwan Endemic Species Research Institute (TESRI) garden, in Nantou County, central Taiwan (Table 1). Their identities were verified by Dr. Chih-Hui Chen at the TESRI garden. After authentication, herbarium voucher specimens were then deposited in the Graduate Institute of Pharmacognosy, Taipei Medical University, Taiwan.

### Preparation of plant extracts

Dried plant leaves were pulverized and extracted twice with 70% acetone. After filtering, the combined filtrates were concentrated under reduced pressure. The final residues were freeze-dried and stored in a closed container until use. We calculated the yields of plant extracts using the following formula: Yield (%) = (mass of the extract/mass of the dried raw plant material) ×100%.

### Determination of total phenolics

We determined the amount of total phenolics in extracts according to a modified Folin-Ciocalteu method [29]. Briefly, an aliquot of sample solution (250-μL, 2.5 mg/mL) was mixed with 1 N Folin-Ciocalteu reagent (250 μL), a 20% sodium carbonate (Na_2_CO_3_) solution (500 μL), and water (4 mL). After incubation at room temperature for 25 min, the reaction mixture was centrifuged at 5,000 rpm for 10 min. The supernatant was measured at 730 nm using a spectrophotometer. The amount of total phenolics was expressed as gallic acid equivalent (GAE) in milligrams per gram dry plant extract. 

### Cell culture

We obtained human skin fibroblast WS-1 cell from CCRC 6003, Hsinchu, Taiwan. Cells were maintained at 37 °C, 5% CO2 humidified incubator as monolayers in 75 mL culture flasks. WS-1 cells were grown in MEM (Eagle) with 2 mM *L*-glutamine, 0.1 mM non-essential amino acids, and 10% inactivated fetal calf serum, 50 U/mL penicillin and 50 μg/mL streptomycin. We cultured the cells until confluence, and harvested them with trypsin-EDTA. For experiments, WS-1 cells (1x 10^5^ cell/well in 0.5 mL medium) were cultured in 24-well plates. Plant extracts (200-10 μg/mL) were added to the cultures. After incubation for 3 days, the medium was removed. Serum-free-MEM was added, and the culture was incubated one additional 1 day. Culture supernatants were collected and frozen –70^ o^C for further analysis. Cells were used for MTT assay.

For UVB irradiation, WS-1 cells were incubated in 24-well plates and the plant extracts were added to the cells as mentioned earlier. After incubation for 3 days, the medium was removed and the cells were washed with PBS. The cells were UVB-irradiated at doses of 20 mJ/cm^2^ using a UVB cross-linker (Vilber Lourmat). After irradiation, the plates were washed with PBS twice, replaced with serum-free MEM, and incubated for 1 day. After incubation, cells were used for MTT assay, and the supernatants were collected for MMP activity assay.

### MTT assay for cell viability

Mitochondrial dehydrogenase activity, which reduces 3-(4,5-dimethylthiazol-2-yl)-2,5-diphenyl tetrazolium bromide (MTT, Sigma, St. Louis) in active mitochondria to purple formazan, was used to determine cell survival in a colorimetric assay. Briefly, WS-1 cells were cultured in the 24-well plate until confluent. After treatment with different plant extracts for three days, media were removed, and serum-free MEM was added. One day later, we collected supernatants for zymography assay and added 20 μL of MTT to the wells for 4 hours with shaking. Isopropanol (100 μL) was added to the wells, and the measurements were done at 570 nm wavelength on an ELISA plate reader (Emax, Molecular Device). The data were expressed as a proportion of cells not treated with plant extracts. Cell morphology was examined with a microscope. 

### MMP-9 zomography

Analysis of MMP by gelatin zymography was modified as described in [30]. Total proteins accumulated in the cell culture medium from WS-1 cells were measured by Bio-Rad protein assay. Total proteins (0.3 μg/mL) were subjected to 10% SDS-PAGE containing gelatin substrate in a non-reducing condition. After electrophoresis, gels were washed with distilled water containing 2.5% Triton^®^ X-100 for 1 h by gentle shaking. The gels were then incubated at 37 °C for 18 hours in buffer (50 mM Tris-HCl (pH 7.5), 0.2 M NaCl, 5 mM CaCl_2_) and subsequently stained with Coomassie Brillrant Blue. They were then destained in 30% methanol containing 10% glacial acetic acid, and clear bands of protein degradation were visualized. We used the recombinant human MMP-9 (0.5 ng) (Chemicon International Inc. Temecula, CA ) as positive controls. For semi-quantitation of MMPs activity, we scanned photographs of the gel with an imaging densitometer system (Kodac 1D Image Analysis System 3.5). In Figure 1 and Figure 2, (A) Lanes 1-3 represent negative control (H_2_O), rMMP-9 (0.5 ng), medium alone without treatment, M54 (100 μg/mL), M47 (100 μg/mL), M50 (100 μg/mL), M67 (100 μg/mL) and retinoic acid (100 μg/mL); (B) Lanes 1-3 represent negative control (H_2_O), rMMP-9, medium alone without treatment, M61 (100 μg/mL), M55 (10 μg/mL), M80 (100 μg/mL), M81 (100 μg/mL), and retinoic acid (100 μg/mL), respectively. Lower panel represents the semiquantitation the MMP-2 and –9 activities by densitometer. Results represent fold-increase in activity over untreated cells. The results of one representative experiment of three separate experiments are shown.
